# Genome-Wide Association Study of Personality Traits in the Long Life Family Study

**DOI:** 10.3389/fgene.2013.00065

**Published:** 2013-05-08

**Authors:** Harold T. Bae, Paola Sebastiani, Jenny X. Sun, Stacy L. Andersen, E. Warwick Daw, Antonio Terracciano, Luigi Ferrucci, Thomas T. Perls

**Affiliations:** ^1^Department of Biostatistics, Boston University School of Public HealthBoston, MA, USA; ^2^New England Centenarian Study, Section of Geriatrics, Department of Medicine, Boston University School of MedicineBoston, MA, USA; ^3^Division of Statistical Genomics, Washington University School of MedicineSt. Louis, MO, USA; ^4^National Institute on Aging, National Institutes of HealthBaltimore, MD, USA; ^5^Department of Geriatrics, College of Medicine, Florida State UniversityTallahassee, FL, USA

**Keywords:** neo scores, GWAS, family study, gene-environment interaction, longevity

## Abstract

Personality traits have been shown to be associated with longevity and healthy aging. In order to discover novel genetic modifiers associated with personality traits as related with longevity, we performed a genome-wide association study (GWAS) on personality factors assessed by NEO-five-factor inventory in individuals enrolled in the Long Life Family Study (LLFS), a study of 583 families (*N* up to 4595) with clustering for longevity in the United States and Denmark. Three SNPs, in almost perfect LD, associated with agreeableness reached genome-wide significance (*p* < 10^−8^) and replicated in an additional sample of 1279 LLFS subjects, although one (rs9650241) failed to replicate and the other two were not available in two independent replication cohorts, the Baltimore Longitudinal Study of Aging and the New England Centenarian Study. Based on 10,000,000 permutations, the empirical *p*-value of 2 × 10^−7^ was observed for the genome-wide significant SNPs. Seventeen SNPs that reached marginal statistical significance in the two previous GWASs (*p*-value <10^−4^ and 10^−5^), were also marginally significantly associated in this study (*p*-value <0.05), although none of the associations passed the Bonferroni correction. In addition, we tested age-by-SNP interactions and found some significant associations. Since scores of personality traits in LLFS subjects change in the oldest ages, and genetic factors outweigh environmental factors to achieve extreme ages, these age-by-SNP interactions could be a proxy for complex gene–gene interactions affecting personality traits and longevity.

## Introduction

Personality traits have been shown to be associated with important health outcomes and longevity (Terracciano et al., 2008). Previous findings suggest that low levels of neuroticism, high levels of conscientiousness, and high levels of extraversion are associated with reduced mortality (Friedman et al., 1993; Wilson et al., 2004, 2005; Weiss and Costa, 2005; Chapman et al., 2011). Centenarian offspring have lower neuroticism and higher extraversion in comparison to published normative data (Givens et al., 2009). We recently assessed domains of agreeableness, conscientiousness, extraversion, neuroticism, and openness to experience using the NEO-five-factor inventory (NEO-FFI) (Costa and McCrae, 1992) in subjects enrolled in the Long Life Family Study (LLFS): a family-based longitudinal study of longevity and healthy aging (Newman et al., 2011). The analysis replicated the association of low neuroticism and high extraversion with longevity and also confirmed differences in distributions of personality scores at different ages.

Heritability estimates of agreeableness, conscientiousness, extraversion, neuroticism, and openness assessed by the NEO-FFI in 6148 Sardinians ranged from 17 to 33% (Pilia et al., 2006), and a recent genome-wide association study (GWAS) and meta-analysis identified many genetic variants associated with personality traits, although just a few reached levels of genome-wide significance (Terracciano et al., 2010; de Moor et al., 2012). This enrichment of associations suggests that personality traits are likely influenced by many genes in a complex manner, each with small effects.

The association between some personality traits and longevity however triggers the question as to whether additional or different genetic variants in long-lived individuals may be associated with longevity-promoting personality traits such as low neuroticism and high extraversion, and as such could contribute to longer life span and health-span. To test this hypothesis, we conducted a GWAS of five domains of NEO-FFI in subjects from the LLFS and examined the results in the context of other studies, Terracciano et al. (2010) and de Moor et al. (2012), and tested gene-by-environment interactions.

## Materials and Methods

### Study participants and NEO five-factor inventory

#### Long Life Family Study

The LLFS is a study of 583 families demonstrating clustering for longevity and healthy aging living in the United States and Denmark. Study eligibility criteria have been described in detail elsewhere (Newman et al., 2011) and enrollment was conducted between 2006 and 2009 from three study centers in the United States (Boston University, University of Pittsburgh, and Columbia University) and in Denmark at the University of Southern Denmark (Sebastiani et al., 2009; Newman et al., 2011). Potential probands were screened for familial longevity using the Family Longevity Selection Score (FLoSS), which scores a family according to birth-year cohort survival probabilities of the proband and siblings (Sebastiani et al., 2009). Family eligibility criteria were a FLoSS >7.0, a proband and at least one living sibling, who did not have dementia, and at least one offspring also willing to participate. The spouses were enrolled as controls of subjects enrolled for familial longevity. Spouses of the offspring generation were recruited as controls while spouses of the proband generation were recruited only if their biological children were enrolled in the study. Enrollment was closed in 2009, and since 2010 subjects have been followed with annual data collections. Phase 1 of the study refers to the initial data collection when the subjects were enrolled and Phase 2 refers to subsequent annual data collections.

#### NEO-five-factor inventory

The NEO-FFI is a shortened version of the Revised NEO Personality Inventory (NEO PI-R) and the correlation between the two versions range from 0.75 to 0.89 (Costa and McCrae, 1992). The NEO-FFI consists of 60 items, with 12 items for each of the five domains of personality as compared to the NEO PI-R which has 48 items per domain. Each item in the NEO-FFI is scored using a five-item Likert scale of agreement with each statement. During Phase 1 of the LLFS (2006–2009), only the domains neuroticism and conscientiousness of the NEO-FFI were administered to 4938 participants during a phone interview or an in-home interview. During Phase 2 (2010–present) of the LLFS, all five domains of the NEO-FFI were administered by phone or mail to all living and willing participants, and the data were distributed by the data management and coordinating center in two batches: an initial batch of data from 3032 participants (July 2011) (Andersen et al., 2012a), and an additional batch of data from ∼1300 participants (November 2012). Because of some loss at follow-up and deaths of participants, the sample size for the three domains of agreeableness, extraversion, and openness was ∼4400 (Table 1). The way in which we received the data from the data coordinating center created an independent split of the entire data into two non-overlapping data sets that will be used for internal replication, even though individuals in the two batches of the data are correlated. Note that in the analysis of the two data sets, we fully account for this family correlation. Note also that while the proband generation receives a full follow-up every year, the offspring generation receives a full follow-up every 3 years. As a result of this staggered follow-up window, 99.5% of the samples in the second batch are primarily comprised of subjects in the offspring generation.

**Table 1:** **Characteristics of studies**.

	LLFS		NECS	BLSA
N	Discovery (as of February 2012)	4595 (N, C) 2628 (A) 2631 (E) 2612 (O)	244	840
	Additional data (as of November 2012)	1279 (A) 1287 (E) 1276 (O)		
Age		71 (SD 16)	79 (SD 7)	59 (SD 17)
Sex		45% Males	50% Males	54% Males
Array		2.5 Million	610 Quad	550K

*Reported are the characteristics of each cohort (LLFS, NECS, and BLSA). Agreeableness, conscientiousness, extraversion, neuroticism, and openness are denoted by A, C, E, N, and O, respectively. Due to the difference in the follow-up windows between the proband and offspring generations, data on additional samples in the domain of agreeableness, extraversion, and openness were released on November 2012*.

#### New England Centenarian Study

The New England Centenarian Study (NECS) is an ongoing study of exceptional longevity^1^ that began in 1995 (Andersen et al., 2012b). Approximately 1500 centenarians, 500 offspring, and 150 spouses of offspring have been enrolled and followed annually. Personality data for this study were collected in 2008 from 244 unrelated offspring of centenarians using the NEO-FFI questionnaire for a study of personality traits and exceptional longevity (Givens et al., 2009).

#### Baltimore Longitudinal Study of Aging

Started in 1958, the Baltimore Longitudinal Study of Aging (BLSA) is an ongoing multidisciplinary study of aging^2^. The community-dwelling volunteers are assessed at scheduled visits, which include personality assessment. A total of 840 subjects were genotyped (using the Illumina 550K array) and completed the NEO-FFI personality questionnaire at least once. The sample included 46% women and had a mean age of 58.5 years (SD = 17) at the baseline personality assessment.

### Genotyping

DNA samples were genotyped at CIDR, and genotypes calls were determined using Illumina recalibrated clusters. The LLFS GWAS SNP data (Illumina Omni 2.5 or 2.5 million SNPs) underwent checking and quality correction centrally at the LLFS data coordinating center (Washington University, St. Louis) where a series of standard procedures were applied. By using GRR package (Abecasis et al., 2001), the GWAS data were checked with the pedigree structure data to verify that the relationships were correct and to avoid sample mismatches. In some cases, direct comparisons of Y and Mitochondrial markers were also applied to verify relationships. Mendelian consistency for all SNPs was assessed with Loki (Heath, 1997) for autosomal markers, PedCheck (O’Connell and Weeks, 1998) for X markers or direct comparison for Y and mitochondrial markers. All Mendel inconsistencies were removed: a sliding threshold depending on minor allele frequency (MAF) was set and if a SNP had inconsistencies below that threshold, the SNP was set to missing in all families with an inconsistency. If the number of families with inconsistencies was above the threshold, the SNP was removed from the analysis. The threshold was set to remove the “worst” ∼0.2% of markers, and ranged from 35 for a MAF of 0.45–0.5 to 1 for a MAF below 0.1. Filters on call rates both by SNP and by individual were also applied as follows: Individuals with a call rate below 97.5% and SNPs with a call rate below 98% had genotype data removed. These steps resulted in 18 individuals and 86,233 autosomal SNPs being removed, as well as 153,363 Mendel inconsistencies set to missing in the families in which they occurred. Approximately two million autosomal SNPs were judged fit for analysis after this filtering.

The NECS DNA samples were genotyped at Boston University using the Illumina Human610-Quad SNP array, with ∼600,000 SNPs. All samples genotyped at Boston University were processed according to the manufacturer’s protocol and BeadStudio Software was used to make genotype calls utilizing the Illumina pre-defined clusters. Samples with less than a 95% call rate were removed and SNPs with a call rate <97.5% were re-clustered. After re-clustering, SNPs with call rates >97.5%, cluster separation score >0.25, excess heterozygosity between −0.10 and 0.10, and MAF >5% were retained in the analysis. We also removed samples with inconsistent sex defined by heterozygosity of the X chromosome that was not consistent with the sex recorded in the database.

### Imputation

In LLFS, imputation of un-typed genotypes was performed using MACH (version 1.0.16) (Li et al., 2010) for pre-phasing the genotypic data and MINIMAC (version May 29, 2012) (Howie et al., 2012) for actual imputation with 1000HG genotypic data (version 2010–2011 data freeze, 2012-03-04 haplotypes) including all races as a reference panel. A number of filters before imputing were implemented in the LLFS genotypic data by removing markers that had MAF <1%, HWE *p*-value <10^−6^, if LLFS SNPs alleles mismatched with those of 1000HG, and not present in the 1000HG panel. As a result, 38,045,518 variants were imputed and only those with *r*^2^ > 0.3 were used for analysis.

### Statistical analysis

Prior to all analyses, raw scores of NEO-FFI in the LLFS and NECS subjects were transformed into sex-specific standardized *T*-scores with mean = 50 and standard deviation of 10 using the sex-specific means and standard deviations in Table B-4 on page 78 of the NEO PI-R manual (Table S1 in Supplementary Material) (Costa and McCrae, 1992). *T*-scores between 45 and 55 represent normal values, while *T*-scores <45 represent lower than normal, and *T*-scores >55 represent higher than normal values. Lack of departure of the *T*-score from normality was verified by using the Kurtosis test as reported in Andersen et al. (2012a).

### Heritability

Heritability estimates (Table S2 in Supplementary Material) were obtained using variance components analysis implemented in Sequential Oligogenic Linkage Analysis Routines (SOLAR) (Almasy and Blangero, 1998). Under variance components analysis, the total phenotypic variance can be modeled as a sum of an additive genetic component and a non-additive genetic component consisting of environmental factors and measurement errors. The narrow-sense heritability is estimated as the ratio of additive genetic variance to the total phenotypic variance. For each domain, covariates included sex, field centers, and significant polynomial terms of age.

### Discovery

Only subjects with completed questionnaires and genotype data (including spouses) and with Caucasian origins were included in the discovery analysis in the LLFS (agreeableness: *n* = 2628; conscientiousness: *n* = 4590; extraversion: *n* = 2631; neuroticism: *n* = 4595; openness: *n* = 2612). This discovery set comprised data released in February 2012 (batch 1). Again, due to the difference in the data collection between the proband and offspring generations, the additional data we received on November 2012 (batch 2) were an independent split of the entire data and were used to replicate the top findings from the discovery set in the domains of agreeableness, extraversion, and openness. There were 2759 participants, who had repeated measures on conscientiousness and neuroticism, and the average time interval between the repeated measures was 2.6 years. The agreement between repeated measures of conscientiousness and neuroticism was estimated using the Spearman correlation coefficient (0.67 and 0.66, respectively) and repeated measures were summarized by the average scores and the average ages. No significant trend between age and difference between the two domains was observed, as reported in Andersen et al. (2012a). The association between the five domains of personality and the genotype for each SNP was tested in a linear mixed model with random effects per subject. The random effects were modeled as a multivariate normal distribution with zero mean vector and variance-covariance matrix proportional to the kinship matrix to fully account for familial relations. Covariates included sex, field centers, and significant polynomial terms of age. Significant polynomial terms of age were searched using the model search strategy described in Andersen et al. (2012a). Analyses incorporating the top 10 principal components were also conducted, but the results did not change substantially with this adjustment. All GWAS analyses were performed with R statistical software (version R.2.14) using the “kinship” package. The additive genetic model, which codes the SNP genotype as the number of minor alleles (0, 1, 2), was assumed. SNPs with MAF greater 5% and genotype count >2 were used. To correct for multiple testing, the genome-wide significance threshold of 10^−8^ was used. Table 2 shows the SNPs that reached genome-wide significance. To assess the chance of a false positive association, 10,000,000 permutation tests were performed for the SNPs that reached genome-wide significance.

**Table 2:** **Genome-wide significant SNPs in the GWAS of LLFS**.

Domain	SNP	Chr	Gene	CA	CAF	LLFS						NECS		BLSA	
						Initial sample		Additional sample		Full sample					
						Beta	P	Beta	P	Beta	P	Beta	P	Beta	P
Agree	rs9650241	8	–	G	0.086	2.89	1.65E−09	1.35	4.11E−02	2.40	8.12E−10	–	–	−0.4	0.21
Agree	rs2701448	8	–	A	0.087	2.87	1.80E−09	1.35	4.04E−02	2.39	9.46E−10	–	–	–	–
Agree	kgp6080058	8	–	A	0.087	2.85	2.44E−09	1.29	4.78E−02	2.35	1.57E−09	–	–	–	–

*CA, coded allele*.

*CAF, coded allele frequency*.

Reported are the three genome-wide significant (p < 10^−8^) SNPs in the discovery (LLFS) and their results in the three replication cohorts (additional sample in LLFS, NECS, and BLSA). Only rs9650241 is found in the BLSA. SNPs that could not be tested are indicated by “–.”

#### Replication in NECS and BLSA

SNPs with *p*-value <10^−5^ in the discovery set were sought for replication in the NECS (*n* = 197) and BLSA (*n* = 848). We defined replication as SNPs having *p*-value <0.05 in the replication cohort and consistent direction of effects as in the discovery set. In the NECS, the association between the five domains and the genotype for each SNP was tested using linear regression analysis, adjusting for gender and significant polynomial terms of age, where appropriate, in PLINK (Purcell et al., 2007) software (Table S3 in Supplementary Material). SNPs that were not in the 610 Illumina array were replaced by the closest proxy SNPs in strongest linkage disequilibrium (*r*^2^ > 0.8), within a region of 50 kb. Consistency of effects for proxy SNPs was checked by examining the coded allele frequencies of the original SNP and the corresponding proxy SNP. For 53 SNPs we could not find a good proxy. In the BLSA the association analyses were conducted using MERLIN^3^, and age, sex, and principal components were used as covariates (Table S4 in Supplementary Material).

#### Replication of findings from other GWASs

All published results from Terracciano et al. (2010) and de Moor et al. (2012) with *p*-value <10^−4^ and 10^−5^ (available through online Supplementary Material), respectively, were tested in the LLFS using the full sample data as of November 2012 and NECS sets. The intention of this replication test was to examine whether any variants that were shown to be significantly associated with personality traits in the previous GWASs also exhibit significant associations in the LLFS and/or NECS. In the case of non-matching SNPs, imputed dosages were used for the LLLF set. The lowest *r*^2^, a measure of correlation between the imputed genotype and true genotype, was 0.74 for the SNPs we tested. Proxy SNPs in the NECS set were searched within the region of up to ±50,000 base pairs and *r*^2^ > 0.8. Again, consistency of effects for proxy SNPs was checked by examining the coded allele frequencies of the original SNP and the corresponding proxy SNP. Note that only one of the SNPs from Terracciano et al.’s study reached marginal statistical significance in the meta-analysis of de Moor et al.

#### SNP-by-age interaction

In order to identify SNPs whose effects change with participants’ age on each NEO domain, we tested the significance of a SNP × Age interaction term for those SNPs with significant main effects (*p*-value <10^−6^), where age represents the participant’s age at the assessment of NEO Genome-wide testing for interaction would require too much power, as reported in Thomas (2010). Therefore, we chose the *p*-value of 10^−6^ to limit the number of significant testing for interaction to 6, 7, 24, 37, and 7 SNPs respectively for agreeableness, conscientiousness, extraversion, neuroticism, and openness, and have some statistical power. Because of the smaller number of tests, a *p*-value <0.05 was used for statistical significance of the interaction term.

## Results

**Figure 1:**
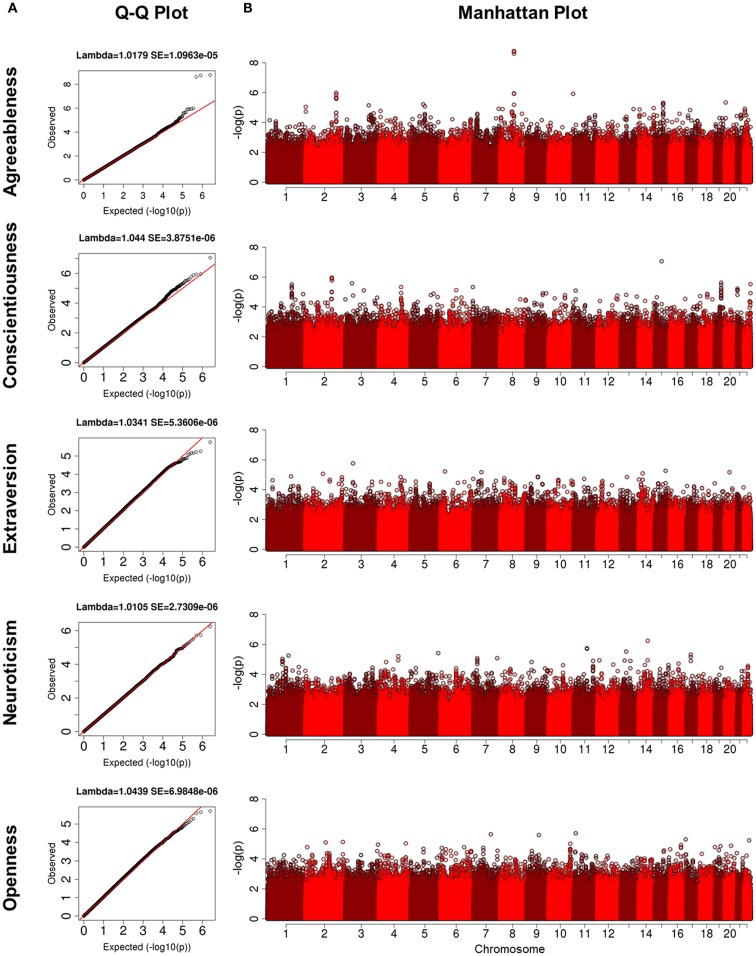
**(A)** Q-Q plots for five domains of NEO-FFI. The y-axis is the quantiles of observed p-values and the x-axis is the quantiles from the expected distribution; **(B)** Manahattan plots for five domains of NEO-FFI. The y-axis is the -log(p) and the x-axis is the genomic locations for each SNP ordered by chromosome and base pair positions.

Characteristics of each cohort are summarized in Table 1. The heritability estimates for the five domains and *p*-values for statistical significance are presented in Table S2 in Supplementary Material and show that all five domains of personality are heritable. Openness was the most heritable (*h*^2^ = 49%), while Agreeableness was the least heritable (*h*^2^ = 18%). The heritability estimates for conscientiousness, extraversion, and neuroticism were 30, 32, and 25%, respectively. With the exception of the higher heritability of openness, the other estimates were comparable to those reported in Pilia et al. (2006). The QQ-plots and Manhattan plots of the GWAS from the LLFS are shown in Figure 1. Three SNPs reached genome-wide significance (Table 2) in the initial GWAS of the LLFS. The top findings (*p*-value <10^−5^) from the LLFS can be found in Table S5 in Supplementary Material (21 SNPs associated with agreeableness; 26 SNPs associated with conscientiousness; 7 SNPs associated with extraversion; 12 SNPs associated with neuroticism; 9 SNPs associated with openness). None of the SNPs that could be tested in the NECS (22 SNPs) or BLSA (24 SNPs) reached statistical significance. Table 3 provides a summary of the replicated results, and Table 4 lists the SNPs in which the genetic effect changes with age. Eighty-one SNPs had significant main effects (*p*-value <10^−6^), and were included in the analysis of significant interactions. There were seven SNPs that had significant interactions (four in extraversion, two in neuroticism, and one in openness). Next, results of specific domains are presented.

**Table 3:** **Summary of SNPs from previous GWAS that replicated in the LLFS and the NECS**.

Domain	SNP	Chr	Gene	CA	CAF	Beta	*P*	CAF	Beta	*P*
					Discovery in SardiNIA			Replication in LLFS		
Agreeableness	rs7042201	9	–	T	0.22	0.14	5.80E−05	0.26	0.51	0.04
Agreeableness	rs382847	9	–	C	0.39	−0.13	3.90E−05	0.26	−0.53	0.04
Agreeableness	rs3204145	9	*IKBKAP*	T	0.16	0.15	5.30E−05	0.19	0.55	0.05
Agreeableness	rs6484998	11	*GALNTL4*	C	0.18	−0.15	2.30E−05	0.18	−0.66	0.02
Agreeableness	rs7121652	11	*GALNTL4*	T	0.17	−0.15	4.50E−05	0.19	−0.62	0.03
Extraversion	rs17147371	11	*PACS1*	C	0.18	−0.17	4.00E−05	0.19	−0.66	0.04
Neuroticism	rs2039528	1	*PTPRF*	G	0.39	−0.14	1.60E−05	0.35	−0.46	0.02
Neuroticism	rs11210864	1	*PTPRF*	A	0.38	−0.14	2.20E−05	0.32	−0.48	0.02
Neuroticism	rs10890251	1	*PTPRF*	C	0.38	−0.14	2.20E−05	0.33	−0.44	0.03
Neuroticism	rs6687571	1	*PTPRF*	A	0.39	−0.13	3.60E−05	0.35	−0.46	0.02
Neuroticism	rs2926458	10	*SORCS3*	T	0.07	0.24	6.90E−05	0.07	0.87	0.03
Neuroticism	rs1606865	12	*TMEM16D*	G	0.4	0.13	9.30E−05	0.41	0.53	0.007
					Discovery in SardiNIA			Replication in NECS		
Agreeableness	rs2202069	15	–	T	0.35	−0.12	3.40E−05	0.32	−2.62	0.02
Neuroticism	rs1421989	5	–	C	0.08	−0.24	1.94E−05	0.16	−3.43	0.03
Neuroticism	rs7317522	13	–	T	0.45	0.12	7.28E−05	0.45	2.39	0.02
					Discovery in de Moor et al. (2012)			Replication in NECS		
Neuroticism	rs12513013	4	*SHROOM3*	C	–	0.45	9.70E−06	0.31	2.73	0.02
Neuroticism	rs7212729	17	*BCAS3*	G	–	0.63	6.10E−06	0.16	4.34	0.005

*CA, coded allele*.

*CAF, coded allele frequency*.

**Table 4:** **SNPs with significant interaction with age**.

Domain	SNP	Chr	Gene	CA	CAF	SNP			Age			SNP × Age		
						Beta	SE	P	Beta	SE	P	Beta	SE	P
Extra	rs79926910	20	KIAA1755	G	0.37	7.48	1.46	3.02E−07	−0.16	0.019	4.54E−17	−0.092	0.019	1.01E−06
Extra	rs877600	20	KIAA1755	A	0.37	7.69	1.45	1.32E−07	−0.16	0.019	1.63E−16	−0.095	0.019	4.75E−07
Extra	rs1205452	20	KIAA1755	C	0.37	7.27	1.46	6.70E−07	−0.16	0.019	1.06E−16	−0.090	0.019	2.23E−06
Extra	rs11258100	10	CCDC3	T	0.09	−11.86	2.42	9.85E−07	−0.26	0.015	1.46E−65	0.148	0.031	2.42E−06
Neuro	rs60933298	7	–	A	0.09	7.47	1.49	5.42E−07	−1.48	0.490	0.0026	−0.097	0.021	2.89E−06
Neuro	rs4728985	7	–	C	0.09	7.48	1.46	3.42E−07	−1.50	0.490	0.0022	−0.098	0.020	1.34E−06
Open	rs7817266	8	–	A	0.34	−6.55	1.33	8.96E−07	2.83	0.902	0.0017	0.083	0.017	1.38E−06

*CA, coded allele*.

*CAF, coded allele frequency*.

### Agreeableness

Three SNPs on chromosome 8, which are in almost perfect LD, reached genome-wide significance in the first batch of data and consistent results in the second batch of data in the LLFS with *p* = 0.04 (Table 2). When analyzed using the combined sample (*n* = 3907), the statistical significance increased to a *p*-value of 8.12 × 10^−10^ for rs9650241 (the most significant SNP). However, these SNPs did not replicate in the BLSA (*p*-value for rs9650241 was 0.21, and the other two SNPs were not found in their array). These three SNPs were not found in the NECS. From Terracciano et al.’s (2010) findings on agreeableness (114 SNPs with *p* < 10^−4^), 109 were found in the LLFS GWAS and 5 replicated, and 20 were found in the NECS GWAS and 1 replicated (Table 3). From de Moor et al.’s (2012) findings on agreeableness (14 SNPs with *p* < 10^−5^), 14 were found in the LLFS GWAS, and 2 in the NECS GWAS, but none replicated.

### Conscientiousness

No SNP reached genome-wide significance. SNP rs79732200 on chromosome 15 in the gene *IGDCC3*, reached almost genome-wide significance (*p*-value 9 × 10^−8^). However, this SNP was not found in either NECS or BLSA GWAS. From Terracciano et al.’s (2010) findings on conscientiousness (35 SNPs with *p* < 10^−4^), 31 were found in the LLFS GWAS, and 6 were found in the NECS GWAS, but none replicated. de Moor et al. (2012) identified rs2576037 in *KATNAL2* to be genome-wide significant (4.9 × 10^−8^), but it did not replicate in the LLFS or NECS GWAS (*p* = 0.7 and *p* = 0.5, respectively). From de Moor et al.’s findings on conscientiousness (110 SNPs with *p* < 10^−5^), 109 were found in the LLFS GWAS, and 26 were found in the NECS GWAS, but none replicated.

### Extraversion

No SNP reached genome-wide significance. From Terracciano et al.’s (2010) findings on extraversion (56 SNPs with *p* < 10^−4^), 55 were found in the LLFS GWAS and 7 were found in the NECS GWAS, and 1 replicated in the LLFS GWAS (Table 3). From de Moor et al.’s (2012) findings on extraversion (30 SNPs with *p* < 10^−5^ reported), 30 were found in the LLFS GWAS and 7 were found in the NECS GWAS, but none replicated. Four SNPs had significant main effects term (*p* < 10^−6^) and interaction term (*p* < 0.05) (Table 4). Three of these SNPs are in strong LD and are in the gene *KIAA1755* and 1 SNP in *CCDC3*. The plots in Figure S1 in Supplementary Material show that the changes of the effect of the minor allele (A) in SNP rs877600 in *KIAA1755* for different ages*:* carriers of the AG and GG genotype (green and blue lines) tend to score higher than carriers of the AA genotype (red line) in extraversion at younger ages (approximately <80 years) but this trend is reversed in older ages. However, the estimated score remains within “average values” until age 100. The effect of the age × SNP interaction for rs11258100 in *CCD3* is different: while carriers of the GG genotype (red line) tend to score lower in extraversion at older ages, in carriers of the genotypes TG and GG the score for extraversion appear to be stable across a wide range of ages.

### Neuroticism

No SNP associated with neuroticisms reached genome-wide significance. From Terracciano et al.’s (2010) findings on neuroticism (46 SNPs with *p* < 10^−4^), 44 were found in the LLFS GWAS and 6 replicated; 12 were found in the NECS GWAS and 2 replicated (Table 3). From de Moor et al.’s (2012) findings on neuroticism (36 SNPs with *p* < 10^−5^), 35 were found in the LLFS GWAS, but none replicated; 3 were found in the NECS GWAS and 2 replicated (Table 3). Two SNPs in strong LD had significant main effects (*p* < 10^−6^) and interaction (*p* < 0.05) terms (Table 4). In Figure S1 in Supplementary Material, subjects with CC genotype (two minor alleles) for SNP rs4782985 have higher neuroticism scores at earlier ages from 40 to 70 than subjects with CT or TT genotypes, but their scores decrease below the normative values after the age of ∼80 years, while subjects with CT or TT genotypes retain the normative values at older ages.

### Openness

No SNP associated with openness reached genome-wide significance. From Terracciano et al.’s (2010) findings on openness (62 SNPs with *p* < 10^−4^), 57 were found in the LLFS GWAS and 18 were found in the NECS GWAS, but none replicated. de Moor et al. (2012) reported rs1477268 and rs2032794 in *RASA1* to be genome-wide significant (*p* = 2.8 × 10^−8^ and 3.1 × 10^−8^, respectively). These SNPs did not replicate in the NECS GWAS with *p* = 0.83 and *p* = 0.71, respectively, and did not replicate in the LLFS GWAS with *p* = 0.69 and *p* = 0.73, respectively. From de Moor et al.’s findings on openness (39 SNPs with *p* < 10^−5^), 39 were found in the LLFS GWAS and 14 were found in the NECS GWAS, but none replicated. In the interaction model, 1 SNP had significant main effects term (*p* < 10^−6^) and interaction term (*p* < 0.05) (Table 4). For this SNP (rs7817266), the three genotype groups show similar trend where their openness scores are higher at earlier ages approximately from 50 to 75 and scores are lower at older ages. However, subjects with AA genotype (two minor alleles) remain within the normative values of openness at all ages, while subjects with AG or GG genotype have openness scores below the normal range after the age of 90.

## Discussion

Prior studies have shown that personality traits have a genetic component (Loehlin and Martinb, 2001; Bouchard and McGue, 2003; Pilia et al., 2006) and the heritability estimates derived in the LLFS data confirm these results. Compared to a twin study (Jang and Vernon, 1996) that reported heritability estimates of 41, 53, 61, 41, and 44%, respectively for neuroticism, extraversion, openness, agreeableness, and conscientiousness, LLFS estimates are lower in every domain. Compared to the recent estimates reported by Pilia et al. (2006) from 6148 Sardinians, the estimates of heritability in the LLFS are higher in conscientiousness, extraversion, and openness. The heritability estimate was lower in LLFS for agreeableness, but comparable for neuroticism. These differences may be due to the fact that samples in the two studies are ethnically different (the Sardinian sample was from a genetically isolated population) and phenotypically different. The LLFS is a study of longevity and families were selected for evidence of familial longevity (Newman et al., 2011). Andersen et al. (2012a) showed that all five domains of NEO scores have different distributions at different ages in the LLFS subjects, and this selected population may be enriched for variants that are associated with longevity as well as longevity-promoting personality traits that translate into different heritability estimates.

Intriguingly, the variants associated with agreeableness that reached genome-wide significance in the LLFS did not replicate in the BLSA. The three genome-wide significant SNPs in agreeableness showed consistent results in the two batches of samples of the LLFS. Even though the two data sets are not independent, the fact that these SNPs show significant associations with consistent effects strengthens the validity of this finding. Among the 10,000,000 permutation tests performed, there was 1 permutation that achieved genome-wide significance, which yields an empirical *p*-value of 2 × 10^−7^ (North et al., 2002). The analysis using the combined samples yielded improved statistical significance for the three SNPs and two additional SNPs that almost attained genome-wide significance (rs2587559 with *p* = 4.13 × 10^−8^ and rs2587561 with *p* = 5.27 × 10^−8^), located in the promoter region of *TRPA1*, which is in a close proximity (within 40,000 bp) to the original variants. This result further corroborates the association between agreeableness and this particular region on chromosome 8, which is linked to tolerance to pain (Doehring et al., 2011). The three genome-wide significant SNPs in the initial GWAS of LLFS have a MAF of around 9%, and there are only 18 subjects out of 2622 with the GG genotype for rs9650241 (the most significant SNP). Therefore, it is difficult to assess the true relationship with the additive coding of SNP genotypes. Under dominant coding (dominant for the G allele) for rs9650241, the statistical significance improves to a *p*-value of 2.1 × 10^−10^, compared to 1.7 × 10^−9^ in an additive model. We observed that the median agreeableness in subjects with the G allele is above the normal range (median *T*-score = 56.1). The association of this locus with agreeableness is novel in the LLFS, although it did not replicate in the BLSA and it could not be tested in the NECS. Therefore, there is no evidence on whether the same association exists in the general population or this locus is linked to agreeableness through longevity.

There were a total of 75 SNPs (21 SNPs associated with agreeableness; 26 SNPs associated with conscientiousness; 7 SNPs associated with extraversion; 12 SNPs associated with neuroticism; 9 SNPs associated with openness, Table S5 in Supplementary Material) with *p*-value <10^−5^ in the GWAS of LLFS. Some of these SNPs were in interesting genes. For example, the most significant SNP in neuroticism (rs177389) is a missense mutation in *PAPLN*, which changes the aminoacid MET into ARG. This gene was shown to be linked to suicidal ideation during anti-depressant treatment (Laje et al., 2009). On average, carriers of the T allele scored lower in neuroticism; the median scores for the three genotypes (GG, GT, TT) were 43.8, 42.7, and 41.4, respectively, all of which are below the normative value. This SNP could not be replicated in the NECS and BLSA GWAS. Likewise, many of the top findings from LLFS remain to be replicated by other investigators in a larger sample with evidence of longevity.

Interestingly, the LLFS GWAS also replicated findings from other studies (Terracciano et al., 2010; de Moor et al., 2012). Six SNPs in agreeableness, 10 SNPs in neuroticism, and 1 SNP in extraversion replicated in either the LLFS or NECS GWAS (Terracciano et al., 2010; de Moor et al., 2012), although none of the replicated associations passed the Bonferroni correction (*p* = 0.00015). de Moor et al. reported several SNPs in *ARNTL* to be associated with agreeableness. In both the LLFS and NECS GWAS, a cluster of SNPs in the same gene were significantly associated with agreeableness at α = 0.05 (Table S6 in Supplementary Material). However, these SNPs were not in LD with those reported in de Moor et al. (highest *r*^2^ = 0.234). *ARNTL*, one of the circadian clock genes, is associated with winter depression and seasonal affective disorder (Partonen et al., 2007) as well as alcohol use disorders (Kovanen et al., 2010). These replicated associations strengthen the original findings in Terracciano et al. (2010), de Moor et al. (2012). Lack of replication of the other findings may be due to different ethnicities, social, and environmental factors.

**Figure 2:**
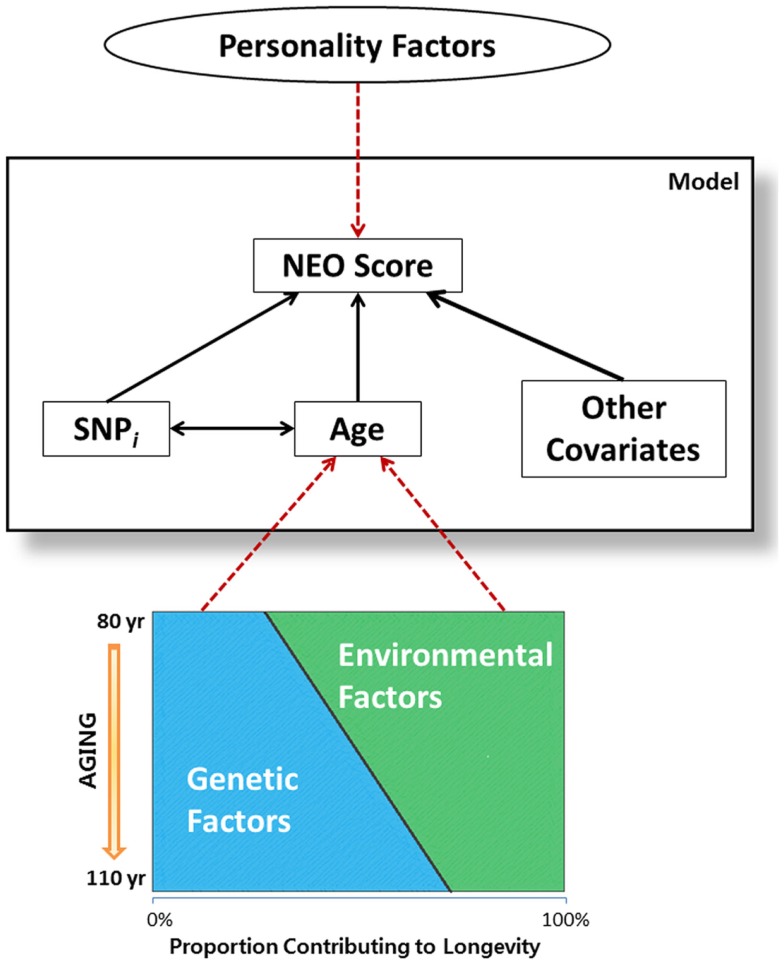
**Graphical representation of hypothesis on gene-gene interactions**. Attributes inside the large rectangular box are the variables included in the statistical model. Age, which is associated with NEO scores, is reflective of both genetic and environmental factors associated with aging. As genetics factors outweigh the environmental factors in individuals enriched for longevity, SNP-by-age interaction term in the model may imply gene-gene interaction which plays important roles in personality traits, as related to longevity.

The significant SNP-by-age interaction terms in Table 4 suggest an interesting hypothesis that it is depicted in Figure 2. It is well known that aging is in part determined by genetic and environmental factors and while genetic factors may explain only 25% of the variability to survive to the mid 1980s (Herskind et al., 1996), the contribution of genes to surviving to older ages is likely much larger (Perls et al., 2000; Sebastiani et al., 2012). Being selected for familial longevity, participants of the LLFS may be enriched for genetic and non-genetic variants that promote longevity, and LLFS subjects who have reached old ages may harbor varying proportions of longevity associated variants. In this hypothetical context, the SNP × age interactions found in this study may actually be surrogates for several gene × gene and gene × environment interactions. This hypothesis is consistent with work in McCrae et al. (2010) that suggested the genetic basis of personality traits is multifactorial, it is likely determined by many interacting genes with individual small effects, and environmental factors are also important factors. However, additional analysis that models the genetic influence on lifespan and personality traits simultaneously is needed to begin to test this hypothesis.

### Limitations

Our ability to replicate the genome-wide significant associations discovered in the LLFS GWAS was limited by the different arrays that were used to genotype the DNA samples from the BLSA and the NECS. In addition, given that five phenotypes are analyzed, a genome-wide significance threshold of 10^−8^ may be too liberal and additional replication is necessary to confirm a role of these discovered variants in personality traits.

## Conclusion

This study is the first GWAS on five major domains of personality traits assessed by NEO-FFI in a sample enriched for longevity. Our results replicated a few loci identified by others, and confirm that effects of each common genetic variant are modest. As with many complex polygenic traits, genetic framework of personality, as related to longevity, seems multifactorial. In particular, genetic variants that promote longevity may interact with other variants in the establishment of personality traits.

## Conflict of Interest Statement

The authors declare that the research was conducted in the absence of any commercial or financial relationships that could be construed as a potential conflict of interest.

## Supplementary Material

The Supplementary Material for this article can be found online at http://www.frontiersin.org/Behavioral_and_Psychiatric_Genetics/10.3389/fgene.2013.00065/abstract

## Footnotes

^1^http://www.bumc.bu.edu/centenarian/

^2^http://www.grc.nia.nih.gov/branches/blsa/blsa.htm

^3^http://www.sph.umich.edu/csg/abecasis/Merlin/
