# Supplementary material for: Genome-Wide Association Study of Personality Traits in the Long Life Family Study
Source: Front Genet. 2013 May 8;4:65. doi: 10.3389/fgene.2013.00065 (PMC3647245; doi:10.3389/fgene.2013.00065)
Supplement: Supplementary file 1 [file 43306_Bae_DataSheet1.DOCX]

**Supplementary Information**

**Genome-wide Association Study of Personality Traits in the Long Life Family Study**

Harold T. Bae, MS^1^, Paola Sebastiani, PhD^1^, Jenny X. Sun, PhD^1^, Stacy L. Andersen, BS^2^, E. Warwick Daw, PhD^3^, Antonio Terracciano, PhD^4, 5^, Luigi Ferrucci, MD, PhD^4^, Thomas T. Perls, MD, MPH^2^

**Supplementary Table 1. Transformation formula for the raw NEO-FFI scores**

| **Transformation Formula** | **Parameters in the Formula** |
| --- | --- |
| $T=10\frac{S- \mu_{n}}{\sigma_{n}}+50$ | S = raw NEO-FFI score |
|  | $\mu_{n}$ = sex-specific population normative mean |
|  | $\sigma_{n}$ = sex-specific population normative standard deviation |
|  |  |
|  |  |

This transformation yields sex-specific standardized T-scores with mean=50 and standard deviation of 10.

**Supplementary Table 2. Heritability Estimates of the five domains of NEO-FFI in subjects of LLFS, in comparison with Heritability Estimates from SardiNIA (**[**10**](#_ENREF_10)**)**

| **NEO Domains** | **Heritability Estimates from LLFS (p-value)** | **Heritability Estimates from SardiNIA (p-value)** |
| --- | --- | --- |
| Agreeableness | 0.176 (1.7x10^-5^) | 0.232 (NS) |
| Conscientiousness | 0.300 (1.7x10^-23^) | 0.204 (<0.01) |
| Extraversion | 0.318 (3.2x10^-15^) | 0.253 (<0.05) |
| Neuroticism | 0.252 (1.7x10^-15^) | 0.254 (<0.01) |
| Openness | 0.486 (4.1x10^-23^) | 0.328 (<0.001) |

Reported are the heritability estimates defined as the ratio between genetic variance and total variance (narrow heritability).

**Supplementary Table 3. Results of Replication of LLFS Findings in NECS**

|  |  |  |  |  | **Discovery in LLFS** | | | | **Replication in NECS** | | | |
| --- | --- | --- | --- | --- | --- | --- | --- | --- | --- | --- | --- | --- |
| **Domain** | **SNP** | **CHR** | **Gene** | **CA** | **CAF** | **Beta** | **SE** | **P** | **CAF** | **Beta** | **SE** | **P** |
| agree | rs11888210 | 2 | ANKRD44 | C | 0.14 | 1.79 | 0.38 | 2.23E-06 | 0.12 | -2.9 | 1.72 | 0.09 |
| agree | rs2587577 | 8 | - | A | 0.16 | 1.64 | 0.36 | 6.53E-06 | 0.2 | 2.18 | 1.28 | 0.09 |
| agree | rs658912 | 5 | ATP6AP1L | T | 0.37 | 1.22 | 0.27 | 6.07E-06 | 0.39 | 0.3 | 1.02 | 0.77 |
| agree | rs8042694 | 15 | COX5A | G | 0.32 | -1.29 | 0.28 | 5.49E-06 | 0.31 | -0.36 | 1.15 | 0.76 |
| consc | rs13397210 | 2 | SCN1A | T | 0.21 | -1.3 | 0.27 | 1.17E-06 | 0.21 | 0.35 | 1.45 | 0.81 |
| consc | rs1841548 | 2 | SCN1A | C | 0.21 | -1.3 | 0.27 | 1.09E-06 | 0.21 | 0.35 | 1.45 | 0.81 |
| consc | rs2781564 | 6 | - | A | 0.44 | 0.97 | 0.22 | 7.50E-06 | 0.39 | 0.73 | 1.2 | 0.54 |
| consc | rs3923603 | 4 | - | A | 0.39 | -1.03 | 0.22 | 4.71E-06 | 0.42 | 0.07 | 1.17 | 0.95 |
| consc | rs6425361 | 1 | - | T | 0.26 | -1.16 | 0.25 | 5.65E-06 | 0.25 | -1.28 | 1.36 | 0.35 |
| consc | rs671000 | 1 | - | T | 0.25 | -1.19 | 0.26 | 4.21E-06 | 0.24 | -0.75 | 1.34 | 0.58 |
| consc | rs7809940 | 7 | - | G | 0.35 | -1.04 | 0.23 | 4.66E-06 | 0.37 | 0.18 | 1.47 | 0.9 |
| consc | rs9822730 | 3 | - | A | 0.2 | -1.2 | 0.27 | 7.83E-06 | 0.18 | -2.72 | 1.58 | 0.09 |
| extra | rs9832895 | 3 | SCN5A | T | 0.48 | 1.38 | 0.29 | 1.73E-06 | 0.48 | 0.86 | 1.14 | 0.45 |
| neuro | rs178244 | 11 | GRM5 | A | 0.48 | -0.91 | 0.19 | 1.82E-06 | 0.48 | -0.57 | 1.08 | 0.6 |
| neuro | rs2173324 | 4 | - | G | 0.38 | 0.89 | 0.2 | 6.08E-06 | 0.35 | -0.3 | 1.3 | 0.82 |
| neuro | rs2189000 | 7 | TWIST1 | G | 0.14 | 1.22 | 0.27 | 8.28E-06 | 0.09 | 2.32 | 1.96 | 0.24 |
| neuro | rs4233429 | 1 | - | T | 0.29 | -0.94 | 0.21 | 9.23E-06 | 0.3 | -0.95 | 1.3 | 0.46 |
| neuro | rs4725336 | 7 | REPIN1 | C | 0.15 | -1.19 | 0.27 | 8.24E-06 | 0.16 | -0.86 | 1.5 | 0.57 |
| open | rs10803639 | 2 | - | A | 0.26 | -1.38 | 0.31 | 7.47E-06 | 0.28 | -0.47 | 1.21 | 0.7 |
| open | rs10831742 | 11 | MICAL2 | G | 0.17 | 1.71 | 0.36 | 1.96E-06 | 0.14 | 0.6 | 1.54 | 0.7 |
| open | rs17039820 | 2 | - | A | 0.07 | 2.31 | 0.52 | 8.02E-06 | 0.08 | -0.57 | 1.98 | 0.77 |
| open | rs2332838 | 4 | - | A | 0.32 | 1.27 | 0.29 | 9.21E-06 | 0.29 | 0.33 | 1.23 | 0.79 |

CA: Coded Allele

CAF: Coded Allele Frequency

Of 75 SNPs with p-value < 10^-5^ in the discovery, only 22 SNPs/proxy SNPs were found in the NECS data. Reported are the replication results for these 22 SNPs with corresponding discovery results.

**Supplementary Table 4. Results of Replication of LLFS Findings in BLSA**

|  |  |  |  |  | **Discovery in LLFS** | | | | **Replication in BLSA** | | | |
| --- | --- | --- | --- | --- | --- | --- | --- | --- | --- | --- | --- | --- |
| **Domain** | **SNP** | **CHR** | **Gene** | **CA** | **CAF** | **Beta** | **SE** | **P** | **CAF** | **Beta** | **SE** | **P** |
| agree | rs10497802 | 2 | ANKRD44 | T | 0.22 | 1.51 | 0.32 | 2.58E-06 | 0.22 | 0.12 | 0.23 | 0.61 |
| agree | rs11888210 | 2 | - | C | 0.14 | 1.79 | 0.38 | 2.23E-06 | 0.11 | 0.17 | 0.32 | 0.6 |
| agree | rs16860099 | 2 | - | C | 0.14 | 1.79 | 0.38 | 2.31E-06 | 0.11 | 0.16 | 0.32 | 0.61 |
| agree | rs16860886 | 2 | ANKRD44 | C | 0.22 | 1.55 | 0.32 | 1.32E-06 | 0.22 | 0.11 | 0.23 | 0.63 |
| agree | rs16860949 | 2 | ANKRD44 | T | 0.23 | 1.5 | 0.32 | 2.58E-06 | 0.22 | 0.12 | 0.24 | 0.6 |
| agree | rs2587559 | 8 | - | A | 0.13 | 1.91 | 0.39 | 1.18E-06 | 0.13 | -0.35 | 0.3 | 0.25 |
| agree | rs2587561 | 8 | - | G | 0.13 | 1.93 | 0.39 | 1.15E-06 | 0.15 | -0.43 | 0.27 | 0.11 |
| agree | rs3789332 | 20 | FERMT1 | G | 0.32 | 1.3 | 0.28 | 4.60E-06 | 0.31 | -0.15 | 0.22 | 0.49 |
| **agree** | **rs9650241** | **8** | **-** | **G** | **0.09** | **2.89** | **0.48** | **1.65E-09** | **0.1** | **-0.4** | **0.31** | **0.21** |
| consc | rs1020852 | 2 | SCN1A | T | 0.21 | -1.28 | 0.27 | 1.68E-06 | 0.19 | 0.51 | 0.35 | 0.15 |
| consc | rs13397210 | 2 | SCN1A | T | 0.21 | -1.3 | 0.27 | 1.17E-06 | 0.12 | 0.49 | 0.49 | 0.32 |
| consc | rs13405797 | 2 | SCN1A | A | 0.21 | -1.29 | 0.27 | 1.30E-06 | 0.19 | 0.44 | 0.35 | 0.21 |
| consc | rs17029245 | 3 | CMTM8 | G | 0.12 | 1.57 | 0.33 | 2.64E-06 | 0.09 | -0.61 | 0.52 | 0.25 |
| consc | rs17801669 | 19 | - | C | 0.24 | -1.17 | 0.25 | 3.31E-06 | 0.25 | 0.24 | 0.33 | 0.47 |
| consc | rs1841548 | 2 | SCN1A | G | 0.21 | -1.3 | 0.27 | 1.09E-06 | 0.19 | 0.44 | 0.35 | 0.21 |
| consc | rs3923603 | 4 | - | T | 0.39 | -1.03 | 0.22 | 4.71E-06 | 0.36 | -0.23 | 0.29 | 0.41 |
| consc | rs671000 | 1 | - | T | 0.25 | -1.19 | 0.26 | 4.21E-06 | 0.24 | -0.04 | 0.33 | 0.9 |
| consc | rs7809940 | 7 | - | G | 0.35 | -1.04 | 0.23 | 4.66E-06 | 0.36 | 0.14 | 0.28 | 0.63 |
| extra | rs9832895 | 3 | SCN5A | T | 0.48 | 1.38 | 0.29 | 1.73E-06 | 0.48 | -0.16 | 0.27 | 0.57 |
| neuro | rs160196 | 11 | GRM5 | C | 0.48 | -0.91 | 0.19 | 1.92E-06 | 0.49 | -0.06 | 0.3 | 0.85 |
| neuro | rs178244 | 11 | GRM5 | A | 0.48 | -0.91 | 0.19 | 1.82E-06 | 0.49 | -0.06 | 0.3 | 0.85 |
| neuro | rs8076171 | 17 | ACCN1 | G | 0.17 | -1.17 | 0.25 | 4.76E-06 | 0.17 | -0.26 | 0.46 | 0.57 |
| open | rs10831742 | 11 | MICAL2 | G | 0.17 | 1.71 | 0.36 | 1.96E-06 | 0.12 | -0.23 | 0.46 | 0.61 |
| open | rs12115280 | 9 | SHC3 | T | 0.13 | -1.95 | 0.41 | 2.61E-06 | 0.11 | -0.46 | 0.53 | 0.39 |

CA: Coded Allele

CAF: Coded Allele Frequency

Of 75 SNPs with p-value < 10^-5^ in the discovery, only 24 SNPs were found in the BLSA data. Reported are the replication results for these 22 SNPs with corresponding discovery results

**Supplementary Table 5. Top Findings (p<10^-5^) from the LLFS**

| **a) Agreeableness** | |  |  |  |  |  |  |  |
| --- | --- | --- | --- | --- | --- | --- | --- | --- |
| **SNP** | **Chr** | **Gene** | **Coded Allele** | **Non-coded Allele** | **Coded AF** | **Beta** | **SE** | **Pval** |
| rs9650241 | 8 | - | G | T | 0.09 | 2.89 | 0.48 | 1.65E-09 |
| rs2701448 | 8 | - | A | G | 0.09 | 2.87 | 0.48 | 1.80E-09 |
| kgp6080058 | 8 | - | A | G | 0.09 | 2.85 | 0.48 | 2.44E-09 |
| kgp12343956 | 2 | - | T | G | 0.14 | 1.86 | 0.38 | 1.04E-06 |
| rs2587561 | 8 | TRPA1 | G | A | 0.13 | 1.93 | 0.39 | 1.15E-06 |
| rs2587559 | 8 | TRPA1 | A | G | 0.13 | 1.91 | 0.39 | 1.18E-06 |
| rs73413011 | 11 | TNNT3 | C | T | 0.25 | -1.48 | 0.3 | 1.22E-06 |
| rs16860886 | 2 | ANKRD44 | C | T | 0.22 | 1.55 | 0.32 | 1.32E-06 |
| rs11888210 | 2 | ANKRD44 | C | T | 0.14 | 1.79 | 0.38 | 2.23E-06 |
| rs16860099 | 2 | ANKRD44 | C | T | 0.14 | 1.79 | 0.38 | 2.31E-06 |
| rs16860949 | 2 | ANKRD44 | T | C | 0.23 | 1.5 | 0.32 | 2.58E-06 |
| rs10497802 | 2 | ANKRD44 | T | G | 0.22 | 1.51 | 0.32 | 2.58E-06 |
| rs3789332 | 20 | FERMT1 | G | A | 0.32 | 1.3 | 0.28 | 4.60E-06 |
| rs7497201 | 15 | MPI | A | G | 0.32 | -1.3 | 0.28 | 4.84E-06 |
| rs8042694 | 15 | COX5A | G | A | 0.32 | -1.29 | 0.28 | 5.49E-06 |
| rs658912 | 5 | ATP6AP1L | T | C | 0.37 | 1.22 | 0.27 | 6.07E-06 |
| rs6495127 | 15 | C15orf17 | C | T | 0.32 | -1.28 | 0.28 | 6.48E-06 |
| rs2587577 | 8 | - | A | G | 0.16 | 1.64 | 0.36 | 6.53E-06 |
| rs1501630 | 3 | SLC9A9 | C | T | 0.41 | 1.2 | 0.27 | 7.13E-06 |
| rs10476618 | 5 | MCTP1 | T | C | 0.45 | 1.18 | 0.26 | 8.40E-06 |
| rs7596238 | 2 | - | A | G | 0.11 | -1.9 | 0.43 | 9.06E-06 |

| **b) Conscientiousness** | | |  |  |  |  |  |  |
| --- | --- | --- | --- | --- | --- | --- | --- | --- |
| **SNP** | **Chr** | **Gene** | **Coded Allele** | **Non-coded Allele** | **Coded AF** | **Beta** | **SE** | **Pval** |
| rs79732200 | 15 | IGDCC3 | A | G | 0.14 | -1.68 | 0.31 | 8.73E-08 |
| rs1841548 | 2 | SCN1A | C | T | 0.21 | -1.3 | 0.27 | 1.09E-06 |
| rs13397210 | 2 | SCN1A | T | C | 0.21 | -1.3 | 0.27 | 1.17E-06 |
| rs13405797 | 2 | SCN1A | A | G | 0.21 | -1.29 | 0.27 | 1.30E-06 |
| rs1020852 | 2 | SCN1A | T | C | 0.21 | -1.28 | 0.27 | 1.68E-06 |
| rs56982446 | 19 | - | G | A | 0.25 | -1.18 | 0.25 | 2.38E-06 |
| rs17029245 | 3 | CMTM8 | G | A | 0.12 | 1.57 | 0.33 | 2.64E-06 |
| rs73173200 | 22 | - | A | G | 0.14 | -1.49 | 0.32 | 3.02E-06 |
| rs535335 | 1 | - | T | C | 0.25 | -1.21 | 0.26 | 3.10E-06 |
| rs17801669 | 19 | - | C | G | 0.24 | -1.17 | 0.25 | 3.31E-06 |
| rs671000 | 1 | - | T | C | 0.25 | -1.19 | 0.26 | 4.21E-06 |
| rs7809940 | 7 | - | G | A | 0.35 | -1.04 | 0.23 | 4.66E-06 |
| rs3923603 | 4 | - | A | G | 0.39 | -1.03 | 0.22 | 4.71E-06 |
| rs73566890 | 19 | - | C | T | 0.24 | -1.15 | 0.25 | 5.20E-06 |
| rs6425361 | 1 | - | T | C | 0.26 | -1.16 | 0.25 | 5.65E-06 |
| rs501915 | 1 | - | C | T | 0.25 | -1.18 | 0.26 | 5.71E-06 |
| rs66528304 | 21 | - | G | A | 0.12 | -1.55 | 0.34 | 5.93E-06 |
| rs56206848 | 19 | - | T | C | 0.24 | -1.14 | 0.25 | 6.95E-06 |
| rs73216895 | 21 | - | G | A | 0.12 | -1.52 | 0.34 | 7.46E-06 |
| rs2781564 | 6 | - | A | C | 0.44 | 0.97 | 0.22 | 7.50E-06 |
| rs73223220 | 21 | - | C | T | 0.12 | -1.53 | 0.34 | 7.56E-06 |
| rs9822730 | 3 | - | A | G | 0.2 | -1.2 | 0.27 | 7.83E-06 |
| rs67483822 | 21 | - | A | G | 0.12 | -1.52 | 0.34 | 7.88E-06 |
| rs1485964 | 11 | MICAL2 | A | C | 0.4 | -0.98 | 0.22 | 8.09E-06 |
| rs55843704 | 19 | - | A | G | 0.2 | -1.19 | 0.27 | 9.71E-06 |
| rs13383881 | 2 | SCN1A | G | A | 0.2 | -1.21 | 0.27 | 9.73E-06 |

| **c) Extraversion** | |  |  |  |  |  |  |  |
| --- | --- | --- | --- | --- | --- | --- | --- | --- |
| **SNP** | **Chr** | **Gene** | **Coded Allele** | **Non-coded Allele** | **Coded AF** | **Beta** | **SE** | **Pval** |
| rs9832895 | 3 | SCN5A | T | C | 0.48 | 1.38 | 0.29 | 1.73E-06 |
| rs12592645 | 15 | - | A | C | 0.18 | -1.79 | 0.39 | 5.44E-06 |
| rs12196515 | 6 | FLJ22536 | A | G | 0.13 | -1.96 | 0.43 | 6.01E-06 |
| rs2598025 | 7 | TXNDC3 | A | C | 0.33 | 1.38 | 0.31 | 6.78E-06 |
| rs77759768 | 20 | TMEM90B | C | A | 0.07 | -2.57 | 0.57 | 6.81E-06 |
| rs12893426 | 14 | - | C | T | 0.24 | 1.54 | 0.34 | 8.03E-06 |
| kgp14702850 | 2 | - | G | A | 0.05 | 2.88 | 0.65 | 8.72E-06 |

| **d) Neuroticism** | |  |  |  |  |  |  |  |
| --- | --- | --- | --- | --- | --- | --- | --- | --- |
| **SNP** | **Chr** | **Gene** | **Coded Allele** | **Non-coded Allele** | **Coded AF** | **Beta** | **SE** | **Pval** |
| rs177389 | 14 | PAPLN | T | G | 0.48 | -0.97 | 0.19 | 5.68E-07 |
| rs178244 | 11 | GRM5 | A | G | 0.48 | -0.91 | 0.19 | 1.82E-06 |
| rs160196 | 11 | GRM5 | C | G | 0.48 | -0.91 | 0.19 | 1.92E-06 |
| rs57565976 | 13 | - | G | C | 0.13 | -1.32 | 0.28 | 2.98E-06 |
| kgp12000284 | 5 | - | T | G | 0.05 | 2.02 | 0.44 | 3.83E-06 |
| rs8076171 | 17 | ACCN1 | G | A | 0.17 | -1.17 | 0.25 | 4.76E-06 |
| rs12045704 | 1 | KIRREL | C | T | 0.06 | 1.88 | 0.41 | 5.55E-06 |
| rs2173324 | 4 | - | G | T | 0.38 | 0.89 | 0.2 | 6.08E-06 |
| rs2881781 | 17 | ACCN1 | A | G | 0.17 | -1.14 | 0.25 | 6.92E-06 |
| rs4725336 | 7 | REPIN1 | C | A | 0.15 | -1.19 | 0.27 | 8.24E-06 |
| rs2189000 | 7 | TWIST1 | G | A | 0.14 | 1.22 | 0.27 | 8.28E-06 |
| rs4233429 | 1 | - | T | G | 0.29 | -0.94 | 0.21 | 9.23E-06 |

| **e) Openness** |  |  |  |  |  |  |  |  |
| --- | --- | --- | --- | --- | --- | --- | --- | --- |
| **SNP** | **Chr** | **Gene** | **Coded Allele** | **Non-coded Allele** | **Coded AF** | **Beta** | **SE** | **Pval** |
| rs10831742 | 11 | MICAL2 | G | A | 0.17 | 1.71 | 0.36 | 1.96E-06 |
| rs78230823 | 7 | - | C | T | 0.13 | 1.85 | 0.39 | 2.28E-06 |
| rs12115280 | 9 | SHC3 | T | C | 0.13 | -1.95 | 0.41 | 2.61E-06 |
| rs72812014 | 17 | NXN | A | G | 0.08 | -2.21 | 0.48 | 5.03E-06 |
| rs7287869 | 22 | UPK3A | G | C | 0.1 | 2.05 | 0.45 | 5.83E-06 |
| rs10803639 | 2 | - | A | G | 0.26 | -1.38 | 0.31 | 7.47E-06 |
| rs17039820 | 2 | - | A | C | 0.07 | 2.31 | 0.52 | 8.02E-06 |
| rs2332838 | 4 | - | A | G | 0.32 | 1.27 | 0.29 | 9.21E-06 |
| rs10901809 | 10 | FAM53B | T | C | 0.31 | -1.29 | 0.29 | 9.76E-06 |

**Supplementary Table 6. SNPs associated with *ARNTL* in the LLFS and NECS GWAS.**

| **SNP** | **CHR** | **Coded Allele** | **Coded Allele Frequency** | **Beta** | **Pval** | **Cohort** |
| --- | --- | --- | --- | --- | --- | --- |
| rs56051850 | 11 | A | 0.21 | 0.73 | 0.029 | LLFS |
| rs10832020 | 11 | C | 0.17 | 0.78 | 0.029 | LLFS |
| rs4757142 | 11 | G | 0.41 | 0.58 | 0.034 | LLFS |
| rs4757145 | 11 | A | 0.40 | 0.56 | 0.044 | LLFS |
| rs969485 | 11 | G | 0.28 | -0.59 | 0.044 | LLFS |
| rs10832030 | 11 | A | 0.15 | -0.73 | 0.050 | LLFS |
| rs7941871 | 11 | C | 0.17 | -3.79 | 0.005 | NECS |
| rs11022693 | 11 | A | 0.31 | 3.04 | 0.007 | NECS |
| rs10831990 | 11 | G | 0.24 | -3.03 | 0.012 | NECS |
| rs10430831 | 11 | G | 0.06 | -4.89 | 0.030 | NECS |


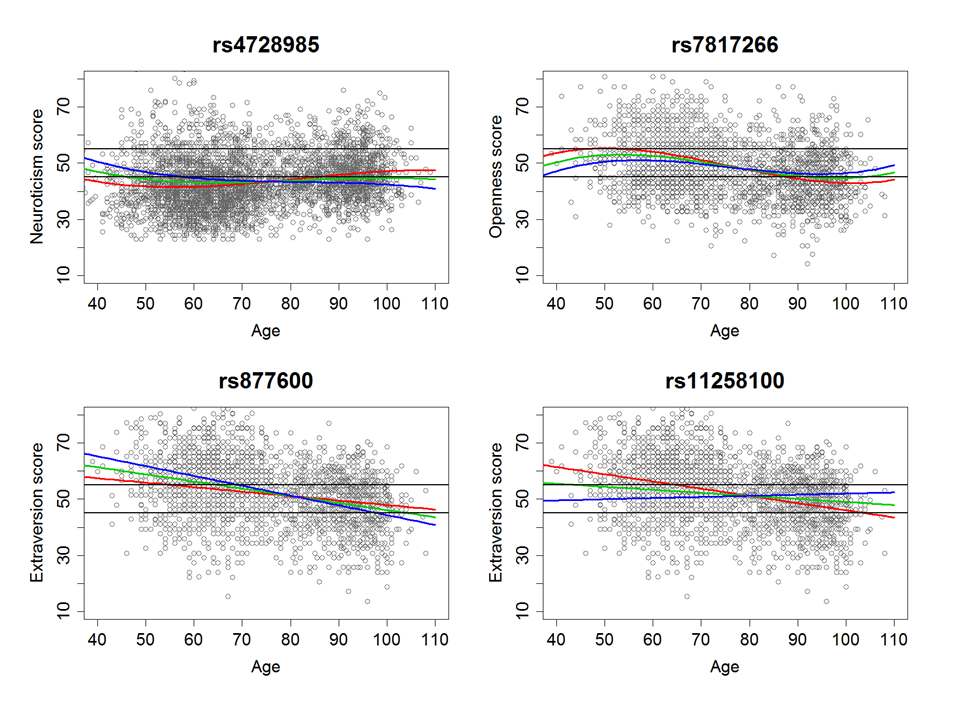


**Supplementary Figure 1. Scatter plots of NEO scores for SNPs with Significant SNP-by-age Interaction**. The y-axis reports the T-score for the NEO domains and the x-axis reports the age at the assessment of NEO-FFI. The red, green, and blue lines represent fitted values for individuals with 0, 1, and 2 minor alleles. The area between the two black, horizontal lines indicates the normal range for T-score values.
